# Comparative Evaluation of IgM Enzyme-Linked Immunosorbent Assay (ELISA) and Real-Time PCR for the Early Diagnosis of Scrub Typhus in Patients With Febrile Illness

**DOI:** 10.7759/cureus.99330

**Published:** 2025-12-15

**Authors:** Pinky Sherawat, Nilofer Khayyam, Shiv Prakash Sharma, Jagveer Singh, Gaurav Dalela

**Affiliations:** 1 Microbiology, Rajasthan University of Health Sciences College of Medical Sciences (RUHS-CMS), Jaipur, IND; 2 Preventive Medicine, Rajasthan University of Health Sciences College of Medical Sciences (RUHS-CMS), Jaipur, IND; 3 Pediatric Neurology, Cradle Children Hospital, Jaipur, IND

**Keywords:** early diagnosis, febrile illness, igm elisa, orientia tsutsugamushi, real-time pcr, scrub typhus

## Abstract

Introduction: Scrub typhus, caused by *Orientia tsutsugamushi*, is a major cause of acute undifferentiated febrile illness (AUFI) in India. Timely diagnosis is challenging due to the non-specific clinical presentation. While IgM enzyme-linked immunosorbent assay (ELISA) is widely used, molecular methods like real-time PCR (RT-PCR) offer the potential for early detection. This study compared the performance of IgM ELISA and RT-PCR in diagnosing scrub typhus in patients with febrile illness.

Methods: A cross-sectional study was conducted from January to April 2024. Blood samples were collected from 156 patients with febrile illness of less than 14 days. All samples were tested for scrub typhus IgM ELISA. ELISA-positive samples and a random selection of ELISA-negative, clinically suspected samples were subjected to RT-PCR for confirmation.

Results: Of the 156 patients, 46 (29.5%) were positive by IgM ELISA. Among these 46 ELISA-positive cases, 15 (32.6%) were positive by RT-PCR. A stark contrast was observed when analyzed by fever duration. Among the 15 RT-PCR positive cases, 100% had a fever duration of less than seven days. In contrast, only 17 (37.0%) of the 46 IgM ELISA-positive cases presented within the first seven days of fever (p<0.001). The 31 IgM ELISA-positive but RT-PCR-negative cases were predominantly from the later febrile phase (seven to 14 days).

Conclusions: RT-PCR is a superior tool for the early diagnosis of scrub typhus within the first week of fever, while IgM ELISA becomes more reliable thereafter. A diagnostic approach utilizing RT-PCR in the initial febrile phase and serology in the later phase can significantly improve the accuracy and timeliness of scrub typhus diagnosis.

## Introduction

Scrub typhus is a life-threatening zoonotic disease caused by the obligate intracellular bacterium *Orientia tsutsugamushi* and transmitted through the bite of infected chigger mites [1]. It is a significant public health concern across the "tsutsugamushi triangle," which includes vast regions of Asia, and is re-emerging as a leading cause of acute undifferentiated febrile illness (AUFI) in India [2,3]. The clinical presentation is often non-specific, characterized by fever, headache, myalgia, and, in some cases, an eschar; however, the latter is highly variable and frequently absent, making clinical diagnosis unreliable [4].

The diagnostic challenges lead to underreporting, delayed treatment, and increased risk of severe complications, including multi-organ dysfunction [5]. The gold standard diagnostic test, the immunofluorescence assay (IFA), is technically demanding, expensive, and not readily available in resource-limited settings endemic to the disease [6]. Consequently, the IgM enzyme-linked immunosorbent assay (IgM ELISA) has become a widely used serological alternative due to its relative ease and cost-effectiveness [7].

However, a significant limitation of serological tests is their dependence on the host's antibody response, which may not be detectable in the early acute phase of the infection [8]. In contrast, molecular techniques like real-time PCR (RT-PCR) can detect bacterial DNA directly, offering the potential for a definitive diagnosis in the initial days of illness [9]. While several studies have evaluated these tests individually, there is a need for clear, practical guidance on their optimal use based on the stage of illness in clinical settings.

This study aimed to comparatively evaluate the diagnostic performance of IgM ELISA and RT-PCR for scrub typhus and to correlate their efficacy with the duration of febrile illness.

## Materials and methods

Study design and population

A laboratory-based, cross-sectional study was conducted over four months (January to April 2024) at the Department of Microbiology, Rajasthan University of Health Sciences College of Medical Sciences (RUHS-CMS), Jaipur, India. After obtaining ethical approval (RUHS-CMS/Ethics Comm./2023/288), 156 patients of all age groups and both sexes presenting with acute undifferentiated fever of less than 14 days were enrolled. Written informed consent was obtained from all participants.

Inclusion and exclusion criteria

All patients with a fever of unknown origin lasting less than 14 days and patients who provided written informed consent were included in the study. In the case of illiterate persons, a witness signed the consent form. Patients who did not provide consent were excluded from the study.

Sample collection and processing

From each patient, 7 mL of venous blood was collected (2 mL in a plain vial and 5 mL in an ethylenediaminetetraacetic acid (EDTA) vial). Serum separated from the plain vial was used for IgM ELISA testing. The whole blood from the EDTA vial was stored at -20°C for subsequent DNA extraction and RT-PCR.

Laboratory methods

Serum samples were tested for scrub typhus IgM antibodies using the commercially available Scrub Typhus Detect™ IgM ELISA system (InBios International, Inc., USA), following the manufacturer's instructions. The optical density (OD) was measured at 450 nm, and a value of ≥0.5 was considered positive. Genomic DNA was extracted from whole blood samples of all IgM ELISA-positive patients and a random selection of IgM ELISA-negative, clinically suspected cases using the TRUPCR® Blood DNA Extraction Kit (3B BlackBio Biotech India Ltd., Bhopal, India). The extracted DNA was then amplified using the TRUPCR® Scrub Typhus Detection Kit (RT PCR-based detection) targeting *O. tsutsugamushi*-specific genes. A cycle threshold (Ct) value of ≤40 was considered positive.

Statistical analysis

Data were entered into a Microsoft Excel (Microsoft Corporation, Redmond, Washington, United States) spreadsheet and analyzed. Descriptive statistics were presented as frequencies and percentages. The chi-square test was used to compare the proportions of test positivity based on fever duration. A p-value of less than 0.05 was considered statistically significant.

## Results

During the study period, 156 patients with febrile illness were tested. The demographic and etiological profile of the febrile cohort is summarized in Table 1.

**Table 1:** Demographic and etiological profile of febrile patients (n=156) Baseline characteristics of the study population.

Characteristic		Frequency	Percentage (%)
Total Scrub Typhus (IgM ELISA +)		46	29.5%
Other Etiologies	Dengue (DEN+)	7	4.5%
	Chikungunya (CHIK+)	1	0.6%
	Typhoid (TYP+)	23	14.7%
	Co-infection (ST+DEN)	4	2.6%
	Co-infection (ST+TYP)	11	7.1%
	Undiagnosed	78	50.0%
Gender Distribution (Total Cohort)	Male	80	51.3%
	Female	76	48.7%

Of the 156 febrile patients tested, 46 (29.5%) were positive for scrub typhus by IgM ELISA. These 46 seropositive samples were subjected to RT-PCR for confirmation. A direct comparison revealed that only 15 of the 46 IgM ELISA-positive cases (32.6%) were also positive by RT-PCR. The remaining 31 cases (67.4%) were IgM ELISA positive but RT-PCR negative, indicating a significant discordance between the two tests (Table 2).

**Table 2:** Comparative analysis of IgM ELISA and RT-PCR results (n=46) Direct comparison of test outcomes among IgM ELISA-positive patients. ELISA: enzyme-linked immunosorbent assay; RT: real-time

Test Result	Frequency	Percentage (%)
IgM ELISA Positive / RT-PCR Positive	15	32.6%
IgM ELISA Positive / RT-PCR Negative	31	67.4%

The relationship between test positivity and the duration of fever revealed a critical and statistically significant pattern (p<0.001). All 15 cases that were positive by RT-PCR (100%) presented within the first seven days of fever. In stark contrast, IgM ELISA detected only 17 of its 46 positive cases (37.0%) in this early febrile phase. The majority of IgM ELISA-positive cases (29 out of 46, 63.0%) presented later in the illness, with a fever duration of seven to 14 days. Furthermore, not a single RT-PCR positive case was found in this later phase (Table 3). This clear dichotomy underscores that RT-PCR is most effective during the initial bacteremic phase, while IgM ELISA becomes more reliable after the first week, coinciding with seroconversion.

**Table 3:** Association between test positivity and duration of fever Comparison of test positivity based on fever duration using the chi-square test, demonstrating a significant association (p<0.001) where RT-PCR exclusively detected cases in the early febrile phase. ELISA: enzyme-linked immunosorbent assay; RT: real-time

Fever Duration	IgM ELISA Positive (n=46)	RT-PCR Positive (n=15)	Test Statistic (χ²)	p-value
< 7 days	17 (37.0%)	15 (100.0%)	22.68	<0.001
7-14 days	29 (63.0%)	0 (0.0%)		

## Discussion

Our study provides clear evidence for the differential utility of IgM ELISA and RT-PCR in diagnosing scrub typhus based on the phase of the febrile illness. The finding that 100% of RT-PCR positive cases occurred within the first week of fever underscores its role as an early diagnostic tool. This is physiologically sound, as the bacterial load is expected to be highest during the initial acute bacteremic phase, which precedes the peak humoral immune response [9].

The lower RT-PCR positivity rate of 15 (32.6%) among the 46 IgM ELISA-positive patients can be attributed to several factors. Many patients likely present after the first week of illness, a time when the bacterial DNA may have cleared or fallen below detectable levels, but IgM antibodies are prominent [10]. Furthermore, prior administration of antibiotics before sample collection, a common occurrence in clinical practice, can significantly reduce the bacterial load, leading to false-negative PCR results while leaving the serological response intact [11]. Our data aligns with studies by Kim et al. and Patricia et al., who also reported higher sensitivity for PCR in the early stages and a decline thereafter [9,12].

Conversely, IgM ELISA demonstrated its value in the later stage (seven to 14 days) of the illness, detecting 29 (63.0%) of its positive cases in this period. This reflects the time required for seroconversion and antibody maturation. However, the potential for persistence of IgM antibodies from past infections can lead to false-positive results, which may explain some of the 31 IgM+/PCR- cases in our study [13]. This dichotomy highlights that the tests are not mutually exclusive but are complementary, each informing a different aspect of the infection timeline [14].

The strategic implication is clear. In a region with high scrub typhus prevalence, relying solely on IgM ELISA for patients presenting early in their illness can lead to missed diagnoses and delayed treatment. Our findings support the implementation of a phase-dependent diagnostic algorithm: using RT-PCR for patients with fever for less than seven days and IgM ELISA for those with a longer duration of symptoms. This approach can optimize resource utilization and improve patient outcomes through timely and accurate diagnosis.

Limitations

This study was conducted at a single center over a short, four-month period, which may affect the generalizability of the findings. The sample size for the RT-PCR analysis was limited to IgM ELISA-positive and a subset of negative samples. IFA, the gold standard, was not performed due to resource constraints, which is a common limitation in many endemic settings.

## Conclusions

RT-PCR is highly effective for the early diagnosis of scrub typhus within the first week of fever, while IgM ELISA is more reliable in the subsequent days. An integrated diagnostic strategy, utilizing RT-PCR for acute-phase patients and serology for those in the later phase, can significantly enhance diagnostic accuracy and facilitate prompt treatment, thereby improving patient outcomes in endemic areas.
